# The Association of Parasitic Infections in Pregnancy and Maternal and Fetal Anemia: A Cohort Study in Coastal Kenya

**DOI:** 10.1371/journal.pntd.0002724

**Published:** 2014-02-27

**Authors:** Elizabeth M. McClure, Steven R. Meshnick, Peter Mungai, Indu Malhotra, Christopher L. King, Robert L. Goldenberg, Michael G. Hudgens, Anna Maria Siega-Riz, Arlene E. Dent

**Affiliations:** 1 Department of Epidemiology, Gillings School of Global Public Health, University of North Carolina at Chapel Hill, Chapel Hill, North Carolina, United States of America; 2 Research Triangle Institute, Durham, North Carolina, United States of America; 3 Center for Global Health and Diseases, Case Western Reserve University, Cleveland, Ohio, United States of America; 4 Department of Obstetrics and Gynecology, Columbia University School of Medicine, New York, New York, United States of America; 5 Department of Biostatistics, Gillings School of Global Public Health, University of North Carolina at Chapel Hill, Chapel Hill, North Carolina, United States of America; University of Pennsylvania, United States of America

## Abstract

**Background:**

Relative contribution of these infections on anemia in pregnancy is not certain. While measures to protect pregnant women against malaria have been scaling up, interventions against helminthes have received much less attention. In this study, we determine the relative impact of helminthes and malaria on maternal anemia.

**Methods:**

A prospective observational study was conducted in coastal Kenya among a cohort of pregnant women who were recruited at their first antenatal care (ANC) visit and tested for malaria, hookworm, and other parasitic infections and anemia at enrollment. All women enrolled in the study received presumptive treatment with sulfadoxine-pyrimethamine, iron and multi-vitamins and women diagnosed with helminthic infections were treated with albendazole. Women delivering a live, term birth, were also tested for maternal anemia, fetal anemia and presence of infection at delivery.

**Principal Findings:**

Of the 706 women studied, at the first ANC visit, 27% had moderate/severe anemia and 71% of women were anemic overall. The infections with highest prevalence were hookworm (24%), urogenital schistosomiasis (17%), trichuria (10%), and malaria (9%). In adjusted and unadjusted analyses, moderate/severe anemia at first ANC visit was associated with the higher intensities of hookworm and *P. falciparum* microscopy-malaria infections. At delivery, 34% of women had moderate/severe anemia and 18% of infants' cord hemoglobin was consistent with fetal anemia. While none of the maternal infections were significantly associated with fetal anemia, moderate/severe maternal anemia was associated with fetal anemia.

**Conclusions:**

More than one quarter of women receiving standard ANC with IPTp for malaria had moderate/severe anemia in pregnancy and high rates of parasitic infection. Thus, addressing the role of co-infections, such as hookworm, as well as under-nutrition, and their contribution to anemia is needed.

## Author Summary

International guidelines recommend routine prevention and treatments which are safe and effective during pregnancy to reduce hookworm, malaria and other infections among pregnant women living in geographic areas where these infections are prevalent. Despite their effectiveness, programs to address common infections such as hookworm, schistosomiasis and malaria during pregnancy have not been widely adopted. Hookworm, malaria and other infections have been associated with anemia in children, but the studies on the impact of these infections on anemia in pregnancy have not been as clear. This study was undertaken to evaluate the prevalence of parasitic infections among women attending antenatal care which provided the nationally recommended malaria preventive treatment program in coastal Kenya. At the first ANC visit, more than 70% of women were anemic, nearly one-fourth had hookworm and about 10% had malaria. Women with high levels of hookworm or malaria infections were at risk of anemia.

## Introduction

Anemia affects nearly 25% of all pregnancies worldwide and more than 40% of those in Sub-Saharan Africa [1]. Associated with poor pregnancy outcomes including increased risk of fetal death and preterm birth, maternal anemia also increases risk of maternal mortality associated with obstetric hemorrhage and severe morbidities [1]–[5]. Defined as hemoglobin <11 g/dL, anemia in pregnancy contributes to maternal morbidities and increased risk for mortality associated with conditions such as post-partum hemorrhage [1], [6]. Maternal anemia may also lead to fetal anemia, and, subsequently, to infant anemia as well as long-term childhood morbidities, including impaired neurodevelopmental outcomes [5], [7]–[10].

Although anemia in pregnancy is multi-factorial, poor nutrition and infection are common causes. In Sub-Saharan Africa, soil-transmitted helminthes (STH) including hookworm, urogenital schistosomiasis, and other parasitic infections such as malaria contribute to the high anemia rates in women and young children [11]–[18]. Helminthic infection prevalence of up to 50% has been documented in some regions in Sub-Saharan Africa [19]. Estimates suggest that more than 25% of pregnant women are infected with hookworm, which causes intestinal bleeding and blood loss, and has been most commonly associated with anemia [1], [19]–[23]. In a study of parasitic infection in pregnancy conducted in coastal Kenya from 2000 to 2005, about 32% of women were infected with hookworm, 31% with urogenital schistosomiasis (*Schistosomiasis haematobium*), and almost 43% with malaria (*Plasmodium falciparum*), while more than 46% of women were co-infected [24].

Parasitic infections, including hookworm, may be evaluated by intensity of infection, as measured by the concentration of eggs in the stool [25]. While most morbidity has been seen with high intensity infections, in populations with low iron stores, even low-intensity hookworm infection has been associated with morbidities [25]–[30]. In addition to hookworm, *P. falciparum* malaria increases risk for moderate and severe maternal anemia [11]–[16]. While urogenital schistosomiasis causes adverse health outcomes including anemia, its association with maternal anemia has been less clearly established [27], [28]. Finally, poor nutrition, which contributes to inadequate intake of iron, folate, and other micronutrients, is common in the geographic areas where these parasitic infections are prevalent, and may have an important role in the relationship of infections and anemia [29]–[33].

Many studies have focused on the effects of a single infectious agent on pregnancy outcome and maternal anemia, although a few studies have attempted to understand the relative effects of multiple agents with conflicting results [24], [30], [31]. Fetal anemia has been documented in association with maternal anemia, with rates of 10% to 23% reported in recent studies in Malawi [5], [7] but its association with infection is less well understood.

With preventative treatment during pregnancy with sulfadoxine-pyrimethamine (IPTp-SP) as recommended by the World Health Organization (WHO) in 2004 [34], rates of malaria in pregnancy have decreased [35]–[37]. Thus, other causes of maternal anemia and poor birth outcomes have become increasingly important. Recent trials show that presumptive hookworm treatment reduces the infection rates in pregnancy, although the impact on pregnancy outcomes such as maternal anemia has varied [38]–[40]. Where hookworm infection is endemic, the WHO recommends provision of antihelminthic treatment (e.g., albendazole or other treatments safe during pregnancy) in the second trimester [41]. Furthermore, safe, effective treatment is available to treat urogenital schistosomiasis during pregnancy and endorsed by the WHO [42], [43]. However, for various reasons, the WHO recommendations for hookworm and urogenital schistosomiasis treatment during pregnancy have not been widely implemented [40], [44]. In this study, we sought to ascertain the contributions of parasitic infection to maternal and fetal anemia among a cohort of pregnant women in coastal Kenya.

## Methods

We conducted a secondary analyses of a large, prospective cohort study on fetal immunity conducted in coastal Kenya from 2006–2009. During that period trained study nurses recruited pregnant women between 20 and 32 weeks gestation who were residents of the area for the study at their first antenatal care (ANC) visit at Mswambweni District Hospital, Mswambweni, in Coast Province Kenya. Women who delivered live, term births at the study hospital had follow-up exam completed. At the first ANC visit, blood, stool and urine samples were collected, in addition to maternal anthropometrics, and basic demographic information. All women diagnosed with helminthic infections were treated with albendazole. Women were not treated for urogenital schistosomiasis during pregnancy, but treatment (e.g., praziquantel) was delayed until after delivery, per standard care in Kenya during the study period. All women enrolled in the study received IPTp-SP, one month of iron tables (60 mg/day) and folate (400 ug/day), and multi-vitamins for the duration of their pregnancy per Kenyan national guidelines.

### Ethics Statement

All women provided written informed consent prior to study enrollment. Institutional review board approval was received by Case Western Reserve University, Kenya Medical Research Institute, and the University of North Carolina at Chapel Hill.

### Measures

Blood was drawn at ANC visits to make both thick and thin blood smears at antenatal care and at delivery for maternal peripheral, cord and placental samples. The remaining blood was processed and stored at the laboratory, under temperature controlled conditions, for subsequent PCR analyses. *P. falciparum* malaria was determined by microscopy using the standard Giemsa stain (thick and thin slices) on site and post-study by PCR/Ligase Detection Reaction Fluorescent Microsphere Assay, as previously described [45]. PCR is considered to have high sensitivity to detect malaria parasitemia; however, microscopy, which is more commonly used in clinical settings, is generally considered reliable to detect malaria present in higher concentrations [46]. For this study, as exposures of interest, microscopy was defined as a proxy for high intensity malaria infection and PCR-positive malaria as any malaria infection.

Maternal stool and urine samples were collected at the first ANC visit and at delivery. Study participants brought a morning stool sample to the respective visit and these stool specimens were brought to the laboratory immediately following the collection by the health worker. Stool samples were tested for hookworm infection and other STH (*Ascaris lumbricoides*, *Trichuris trichuria*, *Strongyloides stercoralis*). Approximately one gram of fresh stool specimen was processed and examined by Richtie's concentration method [47]. STH infections were determined by the presence of ova or larva in the stool sample. Burden was also determined by count of parasites/gram. Urine samples were collected and processed by the laboratory immediately following collection. Urine was evaluated for presence of urogenital schistosomiasis (*S. haematobium*) and results expressed as number of eggs/mL. Schistosomiasis was also categorized as light (0–<50 eggs/mL) or moderate (≥50 eggs/mL), according to WHO criteria [42].

Hemoglobin (Hb) levels were measured at the first ANC visit and at delivery by Coulter counter (Beckman Coulter Inc.). Women were classified as anemic (Hb<11 g/dL) and then categorized as being moderately to severely anemic (Hb<9 g/dL), as the primary outcome, and being mildly to non-anemic (Hb≥9 g/dL) according to the WHO classification of anemia [1]. Cord blood hemoglobin levels were also determined and cord (fetal) hemoglobin defined by hemoglobin <12.5 g/dL, as previously defined [7].

Maternal height and weight were taken at the first ANC visit (generally in the second trimester) and body mass index (BMI) calculated as kg/m^2.^ Since pre-pregnancy BMI was unavailable, to assess BMI, low BMI was defined as the lowest 10^th^ percentile for the gestational age at measurement. The overall BMI ranged from 19.5 to 31.4 kg/m2 and the 10^th^ percentile cut-off for GA at measurement ranged from 19.8 to 20.7 kg/m^2^. Trained study nurses interviewed the women at antenatal care visit to obtain key socio-demographic and basic medical history.

### Analyses

The study was a secondary analysis of data collected as part of a larger study to evaluate the association of *in utero* malaria infection on neurodevelopmental outcomes (NCT00314899). Analyses were performed in SAS version 9.3 (SAS Institute, Cary, NC, USA). Descriptive analyses were performed. Parity, gestational age, maternal age, maternal education, and socio-economic status (as measured by monthly household expenditures) were evaluated as potential confounders, based on previous research [14], [17], [19]–[23]. The risk ratios for moderate/severe anemia associated with each of infections evaluated are presented with and without the potential confounders, using a log-binomial regression model. A backward elimination strategy was employed to estimate the adjusted RR of moderate/severe maternal anemia associated with infections and maternal BMI, accounting for the potential confounders. An *a priori* cut-off (p<0.15) was defined for variable to be considered significant and retained in the final regression model. Analyses were restricted to those with complete case information.

## Results

Of the 813 women screened at ANC, 706 (88%) consented women had blood and urine samples available for anemia, malaria, and schistosomiasis evaluation, respectively. Of these participants, 544 (71%) provided stool samples at antenatal care for measurement of STHs and 394 had outcomes at delivery available.

At enrollment at first ANC, the mean gestational age was 24.5 weeks (SD 3.8 weeks). 516 (71%) were anemic (Hb<11 g/dL) and 190 (27%) had moderate to severe anemia (Hb<9 g/dL). For subsequent analyses, moderate/severe anemia was evaluated as the primary outcome of interest. About 19% of the women were <20 years of age, nearly 86% were married, 21% had no formal education, and about 23% were primagravidas (Table 1). In unadjusted analyses, these factors were not associated with increased risk of anemia. Insecticide-treated bednet use, malaria treatment, and iron/folic acid received 3 months prior to the ANC visit were also not associated with moderate/severe anemia risk. Few women (<2%) received anti-helminth treatment prior to first ANC (data not shown).

**Table 1:** Maternal socio-demographic factors by maternal anemia status at first antenatal care visit, cohort of pregnant women in coastal Kenya, 2006–2009.

Characteristic*	Moderate/severe anemia (Hg<9)	No/mild anemia (Hg≥9)	RR (95% CI)†
	N (%)	N (%)	
Number enrolled	190 (27)	516 (73)	–
Maternal age			
20–44	154 (81)	420 (81)	Referent
<20	36 (19)	96 (19)	1.02 (0.75, 1.39)
Marital status			
Married/partner	163 (86)	453 (88)	Referent
Widow/divorced/single	26 (14)	58 (12)	1.05 (0.95, 1.16)
Formal education			
No education	43 (23)	105 (21)	Referent
Primary education	124 (65)	344 (67)	0.99 (0.68, 1.45)
Secondary/higher education	23 (12)	63 (12)	0.98 (0.46, 2.10)
Gravidity			
Multigravida	145 (77)	385 (75)	Referent
Primigravida	42 (23)	128 (25)	0.97 (0.90, 1.04)
Body Mass Index (BMI), m/kg^2^ ‡			
Low BMI	154 (84)	444 (88)	Referent
Normal BMI	30 (16)	62 (12)	1.30 (0.94, 1.81)
Bednet last 3 mos			
Used bednet	134 (71)	386 (75)	Referent
Not used bednet	53 (28)	127 (25)	1.14 (0.87, 1.50)
Malaria treated last 3 mos			
Treatment provided	18 (9)	53 (10)	Referent
No treatment	171 (91)	460 (90)	1.07 (0.70, 1.63)
Folic acid/iron-last 3 mos			
Folic acid/iron	11 (6)	28 (5)	Referent
No folic acid/iron	178 (94)	487 (95)	0.99 (0.96, 1.03)

*Numbers less than total enrolled reflect missing data.

† Unadjusted Risk ratio (RR) and 95% confidence intervals (CI).

‡ Adjusted for gestational age.

The association of demographic characteristics and the prevalence at first ANC of hookworm infection, PCR-positive malaria (*P. falciparum*), and urogenital schistosomiasis (*S. haematobium*) are summarized in Table 2. Risks of *P. falciparum* PCR-positive (RR 2.29, 95% CI 1.38, 3.79), hookworm (RR 1.42, 95% CI 1.02, 1.98), and urogenital schistosomiasis infection (RR 2.25, 95% CI 1.66, 3.07) were higher among those <20 years compared to women ≥20 years. Risks for infection did not differ significantly by maternal education levels. Primigravidity was associated with increased risk of *P. falciparum* PCR-malaria (RR 1.52, 95% CI 1.11, 2.56) and urogenital schistosomiasis (RR 1.51, 95% CI 1.07, 1.78), but not hookworm infection. About one-fourth (25.7%) of the women reported no use of insecticide-treated bednets (ITNs) prior to enrollment, which was associated with increased risk of malaria infection (RR 2.00, 95% CI 1.23, 3.27).

**Table 2:** Maternal characteristics and association with malaria, hookworm and urogenital schistosomiasis infection in pregnancy at first ANC visit, among a cohort of pregnant women, coastal Kenya, 2006–2009.

	Total	*P. falciparum* †		Hookworm*		*S. haematobium*	
	N (%)	n (%)	RR (95% CI)	n (%)	RR (95% CI)	n (%)	RR (95% CI)
Number	706	59		129		119	
Maternal Age							
20–44	574 (81.3)	39 (7.0)	Referent	96 (22.1)	Referent	80 (14.1)	Referent
<20	132 (18.7)	20 (16.0)	2.29 (1.38, 3.79)	33 (31.4)	1.42 (1.02, 1.98)	39 (30.5)	2.25 (1.66, 3.07)
Formal education							
Secondary/higher level	86 (12.2)	6 (7.0)	Referent	9 (15.3)	Referent	12 (14.6)	Referent
Primary education	468 (66.7)	41 (9.1)	1.30 (0.57, 2.97)	86 (23.8)	1.56 (0.83, 2.93)	80 (17.4)	1.18 (0.68, 2.08)
No education	148 (21.1)	12 (8.5)	1.69 (0.32, 8.80)	34 (29.1)	2.43 (0.69, 8.58)	27 (18.2)	1.41 (0.46, 4.32)
Marital status							
Married/partner	616 (88.0)	48 (8.1)	Referent	115 (24.4)	Referent	100 (16.5)	Referent
Widow/divorced/single	84 (12.0)	10 (12.1)	1.41 (0.83, 2.40)	12 (18.7)	0.86 (0.54, 1.36)	18 (21.7)	1.18 (0.77, 1.83)
Gravidity							
Multigravida	529 (75.7)	39 (7.6)	Referent	95 (23.6)	Referent	81 (15.9)	Referent
Primigravida	170 (24.3)	19 (11.6)	1.52 (1.11, 2.56)	33 (24.6)	1.04 (0.76, 1.47)	38 (19.4)	1.51 (1.07, 1.78)
Maternal BMI							
Normal BMI	598 (86.7)	51 (8.5)	Referent	94 (24.8)	Referent	79 (15.9)	Referent
Low BMI	92 (13.3)	12 (8.7)	0.83 (0.35, 2.01)	16 (21.0)	1.08 (0.66, 1.78)	19 (19.4)	1.04 (0.60, 1.78)
Bednet use last 3 mos							
Yes	520 (74.3)	24 (13.9)	Referent	94 (23.6)	Referent	84 (16.2)	Referent
No	180 (25.7)	35 (6.9)	2.00 (1.23, 3.27)	33 (23.9)	1.01 (0.72, 1.43)	34 (19.9)	1.23 (0.86, 1.76)

*Analyses restricted to women with stool sample available at antenatal care visit (n = 544).

† PCR-diagnosed malaria.

Hookworm (23.7%), *P. falciparum* PCR-malaria (10.8%), *S. haematobium* (17.1%), and *T. trichuria* (10.1%) were the most common infections at the first ANC visit. Of women positive for one of these infections, approximately 10% were co-infected, 4% with urogenital schistosomiasis and hookworm, and 2% with either malaria and hookworm or malaria and schistosomiasis, and the remaining with another combination (data not shown). Hookworm intensity ranged from 1 to 1035 eggs/g; thus all were considered ‘light’ according to the WHO criteria (light defined as <1999 eggs/g). To further evaluate whether relative intensity of infection was associated with outcomes, we also classified the highest intensity of infection (≥100 eggs/g) among the cohort as ‘moderate’ infection.

We next examined the risk for moderate/severe maternal anemia at ANC associated with these infections, in unadjusted and adjusted analyses (Table 3). In analyses adjusted for gestational age, primagravid status, and low BMI, moderate/severe anemia was associated with moderate hookworm infection (aRR 2.53, 95% CI 1.62, 3.92), *P. falciparum* PCR-positive and microscopy positive (aRR 1.45, 95% CI 1.01, 2.08 and aRR 1.98, 95% CI 1.17, 3.35, respectively). *S. haematobium* and *T. trichuria*, although common, were not significantly associated with moderate/severe anemia and few had moderate burden of infection. *S. stercoralis* and *A. lumbricodes* were observed in about 1% of women, using a single sample, which may be an under-representation since single-sample fecal assays have low sensitivity, especially for detecting *S. stercoralis* [48]. These infections were not significantly associated with moderate/severe anemia at ANC, in adjusted or unadjusted analyses. Among all infections, only moderate burden hookworm and malaria were associated with moderate/severe anemia.

**Table 3:** Prevalence of parasitic infections in pregnancy and association of moderate/severe maternal anemia (Hg<9) at first ANC visit, among a cohort of pregnant women in coastal Kenya, 2006–2009.

	Infection	Moderate/severe anemia	p-value	Moderate/severe anemia	p-value
	N* (%)	Unadjusted RR (95% CI)		aRR† (95% CI)	
Hookworm					
Absent	415 (76.3)	Referent		Referent	
Present	129 (23.7)	1.15 (0.86–1.55)	0.3	1.17 (0.88, 1.56)	0.3
0–<100 eggs/g	534 (98.2)	Referent		Referent	
>100 eggs/g	10 (1.8)	2.45 (1.60, 3.77)	<0.0001	2.53 (1.62, 3.92)	<0.0001
*P. falciparum*					
PCR-negative	631 (91.1)	Referent		Referent	
PCR-positive	59 (8.6)	1.38 (0.96, 2.00)	0.08	1.45 (1.01, 2.08)	0.05
MS-negative	516 (95.8)	Referent		Referent	
MS-positive	24 (4.6)	2.12 (1.26, 3.57)	0.004	1.98 (1.17, 3.35)	0.01
*S. haematobium*					
Absent	577 (82.9)	Referent		Referent	
Present	119 (17.1)	1.00 (0.92, 1.09)	1.0	1.00 (0.92, 1.10)	0.9
0–<50 eggs/mL	670 (96.3)	Referent		Referent	
≥50 eggs/mL	26 (3.7)	1.15 (0.64, 2.08)	0.6	1.23 (0.68, 2.22)	0.5
*T. trichura*					
Absent	489 (89.9)	Referent		Referent	
Present	55 (10.1)	1.06 (0.70–1.62)	0.8	1.10 (0.73, 1.67)	0.5
0–<100 eggs/g	542 (99.6)	–		–	
≥100 eggs/g	2 (0.4)				
*S. stercoralis*					
Absent	537 (99.1)	Referent		Referent	
Present‡	5 (0.9)	0.68 (0.12–3.94)	0.7	0.43 (0.07, 2.70)	0.4
*A. lumbricodes*					
Absent	535 (98.5)	Referent		Referent	
Present‡	8 (1.5)	0.42 (0.07–2.66)	0.4	0.67 (0.12, 3.86)	0.7

*Numbers differ due to missing values;

† Adjusted for primagravid status, gestational age at ANC, and low maternal BMI;

‡ None of the participants had ≥50 eggs/mL; MS = Microscopy diagnosed malaria.

For those women who delivered live, term births at the study hospital, we evaluated the association between infection at the first ANC visit with maternal and fetal anemia at delivery, as well as the association of infections detected at delivery for those women and their fetuses who had stool (n = 210), or urine and blood samples (n = 394) available at delivery. Women whose births were included were comparable whose births were excluded (exclusion criteria were delivery outside the study hospital, voluntary discontinuance of study participation, lost to follow up, premature delivery, or non-collection of samples), on socio-demographics, maternal characteristics and infection at ANC (malaria, hookworm) (data not shown).

At delivery, 34.2% of the women had moderate/severe anemia and 18.4% of the neonates had fetal anemia (cord Hb<12.5 g/dL). Moderate hookworm burden at the first ANC visit was associated with moderate/severe maternal anemia at delivery (aRR 2.30, 95% CI 1.42, 3.71), but other infections at first ANC visit were not significantly associated with risk of moderate/severe maternal anemia at delivery (Table 4). Fetal anemia was not significantly associated with any of the infections, in adjusted or unadjusted analyses. Of women tested for presence of hookworm, *P. falciparum* malaria (PCR and microscopy) and schistosomiasis at delivery, none of these infections were significantly associated with maternal or fetal anemia at delivery; however, there were insufficient numbers of high-burden hookworm at delivery to test for this association. Furthermore, hookworm infection at ANC was associated with risk of hookworm infection at delivery and *P. falciparum* malaria infection was associated with risk of malaria infection at delivery (p<0.0001 for both).

**Table 4:** Association of infections and maternal and fetal anemia at delivery, coastal Kenya, 2006–2009.

		Maternal moderate/severe anemia (Hb<9 g/dl) at delivery				Fetal anemia (Hb<12.5 g/dl)			
	N† (%)	RR (95% CI)	p-value	aRR (95% CI)*	p-value	RR (95% CI)	p-value	aRR (95% CI)*	p-value
Infection at first ANC									
Hookworm									
Negative	218 (78)	Referent		Referent		Referent		Referent	
Positive	61 (22)	0.76 (0.52, 1.12)	0.2	0.81 (0.55, 1.20)	0.3	1.10 (0.64, 1.90)	0.7	1.13 (0.65, 1.95)	0.6
0–<100 eggs/mL	274 (98)	Referent		Referent		Referent		1.0	
≥100 eggs/mL	5 (2)	2.43 (1.52, 3.90)	0.0002	2.30 (1.42, 3.71)	0.0006	1.91 (0.58, 6.08)	0.3	1.84 (0.58, 5.84)	0.3
*P. falciparum*									
PCR-negative	310 (89)	Referent		Referent		Referent		Referent	
PCR-positive	37 (11)	1.12 (0.45, 2.78)	0.8	1.02 (0.41, 2.54)	1.0	1.58 (0.44, 5.62)	0.5	1.45 (0.43, 4.91)	0.5
MS-negative	330 (95)	Referent		Referent		Referent		Referent	
MS-positive	17 (5)	1.16 (0.47, 2.89)	0.7	1.08 (0.43, 2.75)	0.9	1.22 (0.44, 3.40)	0.7	1.42 (0.50, 3.97)	0.3
*S. haematobium* ‡									
Negative	310 (83)	Referent		Referent		Referent		Referent	
Positive	62 (17)	1.05 (0.92, 1.19)	0.4	1.05 (0.93, 1.20)	0.4	0.75 (0.21, 2.76)	0.7	0.61 (0.17, 2.21)	0.5
Infection at delivery									
Hookworm‡									
Negative	177 (84)	Referent		Referent		Referent		Referent	
Positive	33 (16)	1.07 (0.63, 1.82)	0.8	1.08 (0.64, 1.84)	0.8	1.14 (0.52, 2.50)	0.7	1.10 (0.51, 2.41)	0.5
*P. falciparum*									
PCR-negative	353 (89)	Referent		Referent		Referent		Referent	
PCR-positive	41 (11)	1.43 (0.94, 2.20)	0.09	1.48 (0.97, 2.27)	0.07	1.58 (0.82, 3.01)	0.2	1.60 (0.84, 3.06)	0.2
MS-negative	384 (97)	Referent		Referent		Referent		Referent	
MS-positive	10 (3)	1.35 (0.69, 2.60)	0.4	1.42 (0.74, 2.74)	0.3	1.66 (0.63, 4.36)	0.3	1.69 (0.64, 4.45)	0.3
*S. haematobium* ‡									
Negative	244 (84)	Referent		Referent		Referent		Referent	
Positive	49 (16)	0.98 (0.63, 1.53)	0.9	0.97 (0.62, 1.53)	0.9	0.80 (0.38, 1.66)	0.5	0.74 (0.36, 1.57)	0.4

*Adjusted for primagravid status and low BMI.

† Different denominators reflect missing samples;

‡ insufficient n to assess moderate burden.

MS = Microscopy diagnosed malaria.

In exploratory analyses, we also examined the association of the anemia at ANC with maternal and fetal anemia at delivery among 210 women and their newborns with both measures available. Women with moderate/severe anemia at first ANC visit had increased risk of maternal anemia at delivery (unadjusted RR 3.84, 95% CI 2.94, 4.98). Fetal anemia was also associated with moderate/severe maternal anemia at first ANC visit and moderate/severe maternal anemia at delivery (RR 1.58, 95% CI 1.02, 2.45, p = 0.05; RR 2.75, 95% CI 1.78, 4.24, p<0.001, respectively) (data not shown).

Finally, a multivariate regression model was developed to assess the infections identified as risk factors associated with moderate/severe maternal anemia at ANC. We found that moderate hookworm (aRR 2.37, 95% CI 1.44, 3.91, p = 0.0007) and *P. falciparum* microscopy-positive malaria infection (and aRR 2.06, 95% CI 1.24, 3.44, p = 0.005, respectively) remained significantly associated with moderate/severe maternal anemia at ANC, when adjusting for primigravid status and low maternal BMI (Table 5).

**Table 5:** Factors associated with moderate/severe maternal anemia at first ANC among a cohort of pregnant women, coastal Kenya 2006–2009.

	Maternal moderate/severe (Hb<9 g/dL) anemia at ANC	
	aRR (95% CI)*	p-value
Hookworm (≥100 eggs/g)	2.37 (1.44, 3.91)	0.0007
*P. falciparum* (MS-positive)	2.06 (1.24, 3.44)	0.005
Low BMI	1.25 (0.86, 1.81)	0.2
Primigravida	0.78 (0.54, 1.11)	0.2

*Regression model adjusted for all variables listed and gestational age.

ANC = Antenatal care; MS = microscopy.

## Discussion

Few studies have examined the burden of helminthic infection and under-nutrition in pregnancy on maternal and fetal anemia in malaria-endemic regions. This is now especially important in geographic areas with a declining incidence of malaria found with widespread use of insecticide-treated bednets and IPTp-SP [49]–[51]. In this study of pregnant women at ANC, the prevalence of *P. falciparum* PCR-malaria had fallen to 9% from previous rates of 40% at delivery reported in a similar region in Kenya, prior to widespread IPTp-SP and ITN's [24]. Despite this decline in the malaria rate, 71% of the pregnant women studied were anemic, and overall more than 25% had moderate/severe anemia. Infection with hookworm (24%), and schistosomiasis (17%), which had less significant reductions since the previous study period [24], were also common, although most hookworm infections were light. Both *P. falciparum* malaria as diagnosed by microscopy and moderate hookworm infections at ANC were associated with moderate/severe anemia at the ANC visit, while urogenital schistosomiasis and trichurisis and light infections were not. No association between PCR-detected malaria infections and anemia was found, probably because malaria detected by PCR included many low-level parasitemias. Thus, our findings are consistent with previous studies showing an association with anemia among populations with higher rather than low-intensity parasitic infection [11]. Compared to the first ANC visit, the prevalence and burden of both infections at delivery were lower and neither type of infection was significantly associated with anemia at delivery. The decrease infection rates detected at delivery may be related to restricting analyses to women with term, live births as well as the consequence of the enhanced treatment and care given to this study cohort.

Socio-demographic factors assessed including age, gravidity, education, socio-economic and marital status, and low BMI were not significantly associated with moderate/severe maternal anemia in this cohort. However, the study was conducted among a relatively homogenous community, and thus these disparities may not have been large enough to be detectable. One limitation was that pre-pregnancy BMI and additional measures of under-nutrition were not available for this cohort and thus a more sophisticated assessment of the relationship of nutritional intake and anemia was not possible. Our findings are also consistent with research suggesting that in the context of low socio-demographic status, even light infections such as hookworm and malaria may be associated with anemia [27], [52]; however, further research is needed to address these relationships.

In low-resource areas where hookworm, malaria, and other parasitic infections, in addition to poor nutritional intake, are common, maternal anemia is prevalent and adversely affects the health of both women and their children. In this study, maternal anemia was associated with increased risk of fetal anemia. While we found no significant association of fetal anemia with maternal infection, these results should be interpreted with caution given the restriction to live, term births and the relatively small sample size, reducing our ability to detect differences. While fetal anemia has been less well studied, emerging research suggests that it may also be common in areas with high-burden of infection [5]–[7], [53]. Since fetal and childhood anemia associated with maternal anemia potentially may lead to long-term impaired neurologic function, 8–10, a better understanding of etiology and effects of fetal anemia is important.

Effective, safe treatments are available to prevent and treat hookworm and malaria, both of which were associated with maternal anemia in this study. While numerous studies have evaluated preventative treatment for malaria in pregnancy, fewer have assessed antihelminthic treatment in the context of malaria treatment. Of those that have assessed hookworm, the results suggested that benefit may be most pronounced among women with higher burden of hookworm infection [22]–[24], [54]–[56]. Additionally, few studies have evaluated the roles of multiple infections and under-nutrition in pregnancy and interventions. In a study assessing the role of malaria, hookworm, and nutrition in Uganda, malaria was significantly associated with maternal anemia while hookworm and nutrition were not. The authors speculated that this was in part due to the relatively good nutritional indicators and coverage of helminthic treatment in the region, while malaria prevention strategies were limited [54].

In contrast to the Uganda study, in our study, while all women in this study received antenatal care including IPTp-SP for malaria, treatment for hookworm as indicated, and iron/folic acid, most were not enrolled until after 20 weeks gestation. Thus, even with relatively good antenatal care, treatment was not initiated until the second trimester at which time anemia was prevalent in this cohort. Furthermore, unlike the region where this study was conducted and despite the international recommendations, uptake of treatment for hookworm, malaria, and schistosomiasis in ANC is still low in many parts of Africa [40], [56]. In part, this may relate to perceptions that treatment has not been associated with improved pregnancy outcomes [38], or may be harmful [40]. Given the high prevalence of anemia seen in our study and elsewhere implementation of known effective interventions prior to or early in pregnancy to reduce anemia and ultimately reduce maternal and newborn mortality is needed.

## Supporting Information

Checklist S1 STROBE checklist.
(DOC)

## References

[1] McLean E, Egli I, Cogswell M (ed) (2012). Worldwide prevalence of anaemia 1993–2005: WHO global database on anaemia. Geneva: World Health Organization.

[2] Ouédraogo S, Koura GK, Accrombessi MM, Bodeau-Livinec F, Massougbodji A, Cot M (2012) Maternal anemia at first antenatal visit: prevalence and risk factors in a malaria-endemic area in Benin. Am J Trop Med Hyg 87: 418–424. 22826498 10.4269/ajtmh.2012.11-0706 PMC3435342

[3] Nwizi EN, Iliyasu Z, Ibrahim SA, Galadanci HS (2011) Socio-demographic and maternal factors in anaemia in pregnancy at booking in Kano, Northern Nigeria. African Journal of Reproductive Health 15: 33–41. 22571103

[4] Hartman TK, Rogerson SJ, Fischer PR (2010) The impact of maternal malaria on newborns. Ann Trop Paediatr 30: 271–282. 21118620 10.1179/146532810X12858955921032

[5] Koura GK, Ouedraogo S, Le Port A, Watier L, Cottrell G, et al (2012) Anaemia during pregnancy: impact on birth outcome and infant haemoglobin level during the first 18 months of life. Trop Med Int Health 17: 283–291. 22146105 10.1111/j.1365-3156.2011.02932.x

[6] Huchon C, Dumont A, Traoré M, Abrahamowicz M, Fauconnier A, Fraser W, Fournier P (2013) A prediction score for maternal mortality in Senegal and Mali. Obstet Gynecol 121: 1049–56. 23635742 10.1097/AOG.0b013e31828b33a4

[7] Adediran A, Gbadegesin A, Adeyemo TA, Akinbami A, Osunkalu VO, et al (2011) Haemoglobin and ferritin concentrations in cord blood in a tertiary health centre in Nigeria. Nig Q J Hosp Med 21: 284–9. 23175892

[8] Rogawski ET, Chaluluka E, Molyneux ME, Feng G, Rogerson SJ, Meshnick SR (2012) The effects of malaria and intermittent preventive treatment during pregnancy on fetal anemia in Malawi. Clin Infect Dis 55: 1096–1102. 22767651 10.1093/cid/cis597

[9] Tamura T, Goldenberg RL, Hou J, Johnston KE, Cliver SP, et al (2002) Cord serum ferritin concentrations and mental and psychomotor development of children at five years of age. J Pediatr 140: 165–70. 11865266 10.1067/mpd.2002.120688

[10] Walker SP, Wachs TD, Gardner JM, Lozoff B, Wasserman GA, et al (2007) Child development: risk factors for adverse outcomes in developing countries. Lancet 369: 145–57. 17223478 10.1016/S0140-6736(07)60076-2

[11] Brooker S, Hotez PJ, Bundy DA (2008) Hookworm-related anaemia among pregnant women: a systematic review. PLoS Negl Trop Dis 2: e291. 18820740 10.1371/journal.pntd.0000291 PMC2553481

[12] McClure EM, Goldenberg RL, Dent AE, Meshnick SR (2013) A systematic review of the impact of prevention of malaria in pregnancy on low birth weight and maternal anemia. Int J Gynec Obst 121: 103–9. 10.1016/j.ijgo.2012.12.014 23490427

[13] Uneke CJ (2007) Impact of placental *Plasmodium falciparum* malaria on pregnancy and perinatal outcome in sub–Saharan Africa: II: effects of placental malaria on perinatal outcome; malaria and HIV. Yale J Biol Med 80: 95–103. 18299721 PMC2248298

[14] Ouédraogo S, Bodeau-Livinec F, Briand V, Huynh BT, Koura GK, et al (2012) Malaria and gravidity interact to modify maternal haemoglobin concentrations during pregnancy. Malar J 11: 348. 23088844 10.1186/1475-2875-11-348 PMC3520734

[15] Valea I, Tinto H, Drabo MK, Huybregts L, Sorgho H, et al (2012) An analysis of timing and frequency of malaria infection during pregnancy in relation to the risk of low birth weight, anaemia and perinatal mortality in Burkina Faso. Malar J 11: 71. 22433778 10.1186/1475-2875-11-71 PMC3338396

[16] Degarege A, Legesse M, Medhin G, Animut A, Erko B (2012) Malaria and related outcomes in patients with intestinal helminths: a cross-sectional study. BMC Infect Dis 12: 291. 23136960 10.1186/1471-2334-12-291 PMC3519704

[17] Ouma P, van Eijk AM, Hamel MJ, Parise M, Ayisi JG, Otieno K, et al (2007) Malaria and anaemia among pregnant women at first antenatal clinic visit in Kisumu, western Kenya. Trop Med Int Health 12: 1515–23. 18076560 10.1111/j.1365-3156.2007.01960.x

[18] Bustinduy AL, Parraga IM, Thomas CL, Mungai PL, Mutuku F, et al (2013) Impact of polyparasitic infections on anemia and undernutrition among Kenyan children living in a *schistosoma haematobium*-endemic area. Am J Trop Med Hyg 88: 433–40. 23324217 10.4269/ajtmh.12-0552 PMC3592521

[19] Sousa-Figueiredo JC, Gamboa D, Pedro JM, Fançony C, Langa AJ, et al (2012) Epidemiology of malaria, schistosomiasis, geohelminths, anemia and malnutrition in the context of a demographic surveillance system in northern Angola. PLoS One 7: e33189. 22493664 10.1371/journal.pone.0033189 PMC3320883

[20] Adegnika AA, Ramharter M, Agnandji ST, Ngoa UA, Issifou S, et al (2010) Epidemiology of parasitic co-infections during pregnancy in Lambarene, Gabon. Trop Med Int Health 15: 1204–9. 20636299 10.1111/j.1365-3156.2010.02598.x

[21] Awasthi S, Bundy DAP, Savioli L (2003) Helminthic infections. BMJ 327: 431–3. 12933732 10.1136/bmj.327.7412.431 PMC188497

[22] Roberts T, Gravett CA, Velu PP, Theodoratou E, Wagner TA, Zhang JS, et al (2011) Epidemiology and aetiology of maternal parasitic infections in low- and middle-income countries. J Glob Health 1: 189–200. 23198118 PMC3484768

[23] Hillier SD, Booth M, Muhangi L, Nkurunziza P, Khihembo M, et al (2008) Plasmodium falciparum and helminth coinfection in a semi urban population of pregnant women in Uganda. J Infect Dis 198: 920–927. 18721060 10.1086/591183 PMC2886962

[24] Fairley JK, Bisanzio D, King CH, Kitron U, Mungai P, Muchiri E, et al (2013) Birthweight in offspring of mothers with high prevalence of helminth and malaria infection in coastal Kenya. Am J Trop Med Hyg 88: 48–53. 23166193 10.4269/ajtmh.2012.12-0371 PMC3541745

[25] Montresor A, Crompton DWT, Hall A, Bundy, DAP Savioli L. (1998) Guidelines for the evaluation of soil-transmitted helminthiasis and schistosomiasis at community level. Geneva: WHO.

[26] Yatich NJ, Jolly PE, Funkhouser E, Agbenyega T, Rayner JC, et al (2010) The effect of malaria and intestinal helminth co-infection on birth outcomes in Kumasi, Ghana. Infect Dis Obstet Gynecol 82: 28–34. 10.4269/ajtmh.2010.09-0165 PMC2803505 20064991

[27] Friedman JF, Mital P, Kanzaria HK, Olds GR, Kurtis JD (2007) Schistosomiasis and pregnancy. Trends Parasitol 23: 159–64. 17336160 10.1016/j.pt.2007.02.006

[28] Nour NM (2010) Schistosomiasis: health effects on women. Rev Obstet Gynecol 3: 28–32. 20508780 PMC2876318

[29] Bechir M, Schelling E, Hamit MA, Tanner M, Zinsstag J (2010) Parasitic infections, anemia and malnutrition among rural settled and mobile pastoralist mothers and their children in Chad. Ecohealth 9: 122–3127. 10.1007/s10393-011-0727-5 PMC3415615 22160444

[30] Agu PU, Ogboi JS, Akpoigbe K, Okeke T, Ezugwu E (2013) Impact of Plasmodium falciparum and hookworm infections on the frequency of anaemia in pregnant women of rural communities in Enugu, South East Nigeria. Pan Afr Med J 14: 27. 23503560 10.11604/pamj.2013.14.27.1925 PMC3597852

[31] Woodburn PW, Muhangi L, Hillier S, Ndibazza J, Namujju PB, et al (2009) Risk Factors for helminth, malaria, and HIV infection in pregnancy in Entebbe, Uganda. Plos Neg Trop Dis 3: e473. 10.1371/journal.pntd.0000473 PMC2696595 19564904

[32] Neggers Y, Goldenberg RL (2003) Some thoughts on body mass index, micronutrient intakes and prepregnancy outcome. Journal of Nutrition 133: 1737S–1740S. 12730492 10.1093/jn/133.5.1737S

[33] van Eijk AM, Lindblade KA, Odhiambo F, Peterson E, Rosen DH, Karanja D, et al (2009) Geohelminth infections among pregnant women in rural western Kenya; a cross-sectional study. PLoS Negl Trop Dis 3: e370. 19172184 10.1371/journal.pntd.0000370 PMC2627942

[34] World Health Organization. Updated WHO Policy Recommendation (2012). Intermittent Preventive Treatment of malaria in pregnancy using Sulfadoxine/Pyrimethamine (IPTp-SP). http://www.who.int/malaria/iptp_sp_updated_policy_recommendation_en_102012.pdf

[35] Ouédraogo S, Koura GK, Bodeau-Livinec F, Accrombessi MM, Massougbodji A, et al (2013) Maternal anemia in pregnancy: Assessing the effect of routine preventive measures in a malaria-endemic area. Am J Trop Med Hyg 88: 292–300. 23296448 10.4269/ajtmh.12-0195 PMC3583320

[36] Steketee RW, Campbell CC (2010) Impact of national malaria control scale-up programmes in Africa: magnitude and attribution of effects. Malaria Journal 9: 299. 20979634 10.1186/1475-2875-9-299 PMC2988827

[37] Wilson NO, Ceesay FK, Obed SA, Adjei AA, Gyasi RK, et al (2011) Intermittent preventive treatment with sulfadoxine-pyrimethamine against malaria and anemia in pregnant women. Am J Trop Med Hyg 85: 12–21. 21734118 10.4269/ajtmh.2011.10-0512 PMC3122337

[38] Haider BA, Humayun Q, Bhutta ZA (2009) Effect of administration of antihelminthics for soil transmitted helminths during pregnancy. Cochrane Database Syst Rev 2: CD005547. 10.1002/14651858.CD005547.pub2 19370621

[39] Ndibazzza J, Muhangi L, Akishule D, Kiggundu M, Ameke C, et al (2010) Effects of deworming during pregnancy on maternal and perinatal outcomes in Entebbe, Uganda: a randomized controlled trial. CID 50: 531–539. 10.1086/649924 PMC2857962 20067426

[40] Elliott AM, Ndibazza J, Mpairwe H, Muhangi L, Webb EL, et al (2011) Treatment with anthelminthics during pregnancy: what gains and what risks for the mother and child? Parasitology 138: 1499–1507. 21810307 10.1017/S0031182011001053 PMC3178871

[41] WHO (1994) Report of the WHO informal consultation on hookworm infection and anaemia in girls and women. WHO/CTD/SIP/96.1. World Health Organization, Geneva.

[42] WHO (2002) Prevention and control of schistosomiasis and soil-transmitted helminthiasis: Report of a WHO Expert Committee. Geneva: World Health Organization; 2002. 12592987

[43] WHO (2002) Report of the WHO informal consultation on the use of praziquantel during pregnancy/lactation and albendazole/mebendazole in children under 24 months. Geneva: World Health Organization. WHO/CDS/CPE/PVC/2002.4.

[44] Basra A, Mombo-Ngoma G, Melser MC, Diop DA, Würbel H, et al (2013) Efficacy of Mefloquine intermittent preventive treatment in pregnancy against *Schistosoma haematobium* infection in Gabon: A nested randomized controlled assessor-blinded clinical Trial. Clin Infect Dis 56: e68–75. 23175561 10.1093/cid/cis976

[45] McNamara DT, Kasehagen LG, Grimberg BT, Cole-Tobian J, Collins WE, et al (2006) Diagnosing infection levels of four human malaria parasite species by a polymerase chain reaction/ligase detection reaction fluorescent microsphere-based assay. Am J Trop Med Hyg 74: 413–21. 16525099 PMC3728833

[46] Kattenberg JH, Ochodo EA, Boer KR, Schallig HDFH, Mens PF, et al (2011) Systematic review and meta-analysis: rapid diagnostic tests versus placental histology, microscopy and PCR for malaria in pregnant women. Malaria Journal 10: 321. 22035448 10.1186/1475-2875-10-321 PMC3228868

[47] Ritchie LS (1948) An ether sedimentation technique for routine stool examination. Bulletin of the United States Army Medical Department 8: 326. 18911509

[48] Khieu V, Schär F, Marti H, Sayasone S, Duong S, Muth S, Odermatt P (2013) Diagnosis, treatment and risk factors of Strongyloides stercoralis in schoolchildren in Cambodia. PLoS Negl Trop Dis 7: e2035. 23409200 10.1371/journal.pntd.0002035 PMC3566990

[49] Okiro EA, Hay SI, Gikandi PW, Sharif SK, Noor AM, et al (2007) The decline in paediatric malaria admissions on the coast of Kenya. Malar J 6: 151. 18005422 10.1186/1475-2875-6-151 PMC2194691

[50] Mwangangi JM, Mbogo CM, Orindi BO, Muturi EJ, Midega JT, et al (2013) Shifts in malaria vector species composition and transmission dynamics along the Kenyan coast over the past 20 years. Parasit Vectors 6: 114. 23297732 10.1186/1475-2875-12-13 PMC3544599

[51] Gitonga CW, Edwards T, Karanja PN, Noor AM, Snow RW, et al (2012) Plasmodium infection, anaemia and mosquito net use among school children across different settings in Kenya. Trop Med Int Health 17: 858–70. 22574948 10.1111/j.1365-3156.2012.03001.x PMC3429867

[52] Gyorkos TW, Gilbert NL, Larocque R, Casapía M, Montresor A (2012) Re-visiting *Trichuris trichiura* intensity thresholds based on anemia during pregnancy. PLoS Negl Trop Dis 6: e1783. 23029572 10.1371/journal.pntd.0001783 PMC3441397

[53] Mwinga K, Vermund SH, Chen YQ, Mwatha A, Read JS, et al (2009) Selected hematologic and biochemical measurements in African HIV-infected and uninfected pregnant women and their infants: the HIV Prevention Trials Network 024 Protocol. BMC Pediatrics 9: 49. 19664210 10.1186/1471-2431-9-49 PMC2746190

[54] Muhangi L, Woodburn P, Omara M, Omoding N, Kizito D, et al (2007) Associations between mild-to-moderate anaemia in pregnancy and helminth, malaria and HIV infection in Entebbe, Uganda. Royal Soc Trop Med Hyg 101: 899–907. 10.1016/j.trstmh.2007.03.017 PMC1950430 17555783

[55] Makhoul Z, Taren D, Duncan B, Pandey P, Thomson C, Winzerling J, et al (2012) Risk factors associated with anemia, iron deficiency and iron deficiency anemia in rural Nepali pregnant women. Southwest Asian J Trop Med Public Health 43: 735–46. 23077854

[56] Agu PU, Ogboi JS, Akpoigbe K, Okeke T, Ezugwu E (2013) Impact of Plasmodium falciparum and hookworm infections on the frequency of anaemia in pregnant women of rural communities in Enugu, South East Nigeria. Pan Afr Med J 14: 27. 23503560 10.11604/pamj.2013.14.27.1925 PMC3597852

